# Personal

**Published:** 1917-01

**Authors:** 


### PERSONAL

---

Dr. M. A. Conboy of Buffalo returned from a trip to N. Y. Dec. 11.

---

Dr. Sergeant P. Martin, who has been for the past three years in the Urological Department of the Mayo Clinic, announces that after January 1st, 1917, he will be associated with Dr. James A. Gardner, Electric Building, Buffalo, in the practice of genito-urinary surgery.

---

Dr. James B. Cross of Buffalo announces the removal of his offices to suite 708 Niagara Life Building, Franklin and Mohawk Streets, Buffalo. Practice limited to urology, cystoscopy and genito-urinary diseases.

